# Retraction Note: Rare causes of scoliosis and spine deformity: experience and particular features

**DOI:** 10.1186/s13013-018-0153-3

**Published:** 2018-05-29

**Authors:** Konstantinos C. Soultanis, Alexandros H. Payatakes, Vasilios T. Chouliaras, Georgios C. Mandellos, Nikolaos E. Pyrovolou, Fani M. Pliarchopoulou, Panayotis N. Soucacos

**Affiliations:** 1 1st Department of Orthopaedic Surgery, School of Medicine, University of Athens, “Attikon” Hospital, Rimini 1 Haidari, 12462 Athens, Greece; 2 Department of Orthopaedic Surgery, School of Medicine, University of Ioannina, Panepistemiou Avenue, 45 110 Ioannina, Greece; 3 Department of Surgery, Division of Orthopaedic Surgery, Duke University Medical Center, Durham, NC 27710 USA; 4 4th Department of Internal Medicine, School of Medicine, University of Athens, “Attikon” Hospital, Rimini 1 Haidari, 12462 Athens, Greece

## Retraction

The authors are retracting this article [1] at the request of one of the patients. Consent to publish was obtained but subsequently withdrawn. The article is no longer available online in order to protect the privacy of the individual. The authors apologize to readers for the inconvenience.
